# Mechanical stimulation orchestrates the osteogenic differentiation of human bone marrow stromal cells by regulating HDAC1

**DOI:** 10.1038/cddis.2016.112

**Published:** 2016-05-12

**Authors:** J Wang, C D Wang, N Zhang, W X Tong, Y F Zhang, S Z Shan, X L Zhang, Q F Li

**Affiliations:** 1 Department of Plastic and Reconstructive Surgery, Shanghai Ninth People's Hospital, Shanghai Jiao Tong University School of Medicine, Shanghai 200011, China; 2 The Key Laboratory of Stem Cell Biology, Institute of Health Sciences, Shanghai Institutes for Biological Sciences (SIBS), Chinese Academy of Sciences (CAS) and Shanghai Jiao Tong University School of Medicine, Shanghai 200023, China; 3 Department of Orthopaedics and Traumatology, Prince of Wales Hospital, The Chinese University of Hong Kong, Shatin, New Territories 999077, Hong Kong

## Abstract

Mechanical stimulation and histone deacetylases (HDACs) have essential roles in regulating the osteogenic differentiation of bone marrow stromal cells (BMSCs) and bone formation. However, little is known regarding what regulates HDAC expression and therefore the osteogenic differentiation of BMSCs during osteogenesis. In this study, we investigated whether mechanical loading regulates HDAC expression directly and examined the role of HDACs in mechanical loading-triggered osteogenic differentiation and bone formation. We first studied the microarrays of samples from patients with osteoporosis and found that the NOTCH pathway and skeletal development gene sets were downregulated in the BMSCs of patients with osteoporosis. Then we demonstrated that mechanical stimuli can regulate osteogenesis and bone formation both *in vivo* and *in vitro*. NOTCH signaling was upregulated during cyclic mechanical stretch (CMS)-induced osteogenic differentiation, whereas HDAC1 protein expression was downregulated. The perturbation of HDAC1 expression also had a significant effect on matrix mineralization and JAG1-mediated Notch signaling, suggesting that HDAC1 acts as an endogenous attenuator of Notch signaling in the mechanotransduction of BMSCs. Chromatin immunoprecipitation (ChIP) assay results suggest that HDAC1 modulates the CMS-induced histone H3 acetylation level at the JAG1 promoter. More importantly, we found an inhibitory role of Hdac1 in regulating bone formation in response to hindlimb unloading in mice, and pretreatment with an HDAC1 inhibitor partly rescued the osteoporosis caused by mechanical unloading. Our results demonstrate, for the first time, that mechanical stimulation orchestrates genes expression involved in the osteogenic differentiation of BMSCs via the direct regulation of HDAC1, and the therapeutic inhibition of HDAC1 may be an efficient strategy for enhancing bone formation under mechanical stimulation.

Osteoporosis is a debilitating bone disease that can occur when bone marrow stromal cells (BMSCs) fail to produce a sufficient number of osteoblasts to counteract bone resorption by osteoclasts. The fate of BMSCs is determined based on the integration of chemical (including bone morphogenetic proteins (BMPs), Wnt, MAPKs, and Notch signaling),^1^ spatial and physical signals.^2, 3, 4, 5^ Mechanical stretching has previously been reported to be an important regulator of diverse biological and pathological processes.^6^ It has been convincingly demonstrated that tensile force induces osteogenic differentiation and that compression force induces chondrogenic differentiation.^7, 8^ An analysis of the effects of cyclic mechanical stimulation on the osteogenic differentiation of human MSCs shows that 10% cyclic tensile strain (0.5 Hz) enhances the activity of alkaline phosphatase (ALP) and the rate calcium deposition.^9^ Mechanical stimulation induces osteoblast marker gene expression and the secretion of hormones and growth factors, which affects the differentiation potential of BMSCs and modulates bone remodeling and homeostasis. Clinically, decreases in mechanical loading may result in significant bone loss in weight-bearing (WB) bones and, subsequently, a rapid progression of osteoporosis.^10, 11^ However, a greater understanding of the role of mechanical loading in regulating the differentiation of human BMSCs is required.

In recent years, multiple microRNAs (miRNAs)^12, 13, 14^ and histone modification enzymes^15^ have been found to regulate osteogenic marker gene expression and osteogenesis *in vitro*.^16, 17^ The family of histone deacetylase (HDAC) enzymes comprises at least 18 genes classified into four groups, including class I (HDAC1, HDAC2, HDAC3, and HDAC8) and class II (HDAC4, HDAC5, HDAC6, HDAC7, HDAC9, and HDAC10).^18^ The inhibition of HDAC1 activity typically leads to the activation of transcription. During osteogenesis, total HDAC enzymatic activity is decreased, with a significant reduction in HDAC1 expression. Consistent with this finding, the recruitment of HDAC1 to the promoters of osteoblast marker genes, including osterix (Osx) and osteocalcin (Ocn), is downregulated, whereas histone H3 and H4 are hyperacetylated at those promoters during osteogenic differentiation.^19^ Previous studies have also shown that the suppression of HDAC activity with HDAC inhibitors accelerates osteogenesis.^20^ However, little is known regarding what regulates HDAC expression during osteogenesis. In addition, the functional roles of HDACs in the mechanotransduction of BMSCs have not been well characterized and are, therefore, particularly interesting to study.

BMSCs are simultaneously exposed to chemical and mechanical cues. In this study, we found that HDAC1 was negatively correlated with osteogenic differentiation and bone formation in the BMSCs of patients with osteoporosis, whereas jagged 1 (JAG1)-mediated NOTCH signaling was upregulated. Specifically, mechanical loading directly induced a downregulation of HDAC1 expression, which was involved in the promotion of osteogenic differentiation and bone formation through the targeting of JAG1, a master inducer of osteogenic differentiation.^21, 22, 23^ Our findings also demonstrate that the therapeutic inhibition of HDAC1 may partly rescue osteoporosis caused by mechanical unloading. This study may provide a novel mechanism and potential therapeutic target for enhancing bone formation under mechanical stimulation.

## Results

### Skeletal development and Notch signaling pathways were impaired in BMSCs from patient with osteoporosis

**Figure 1:**
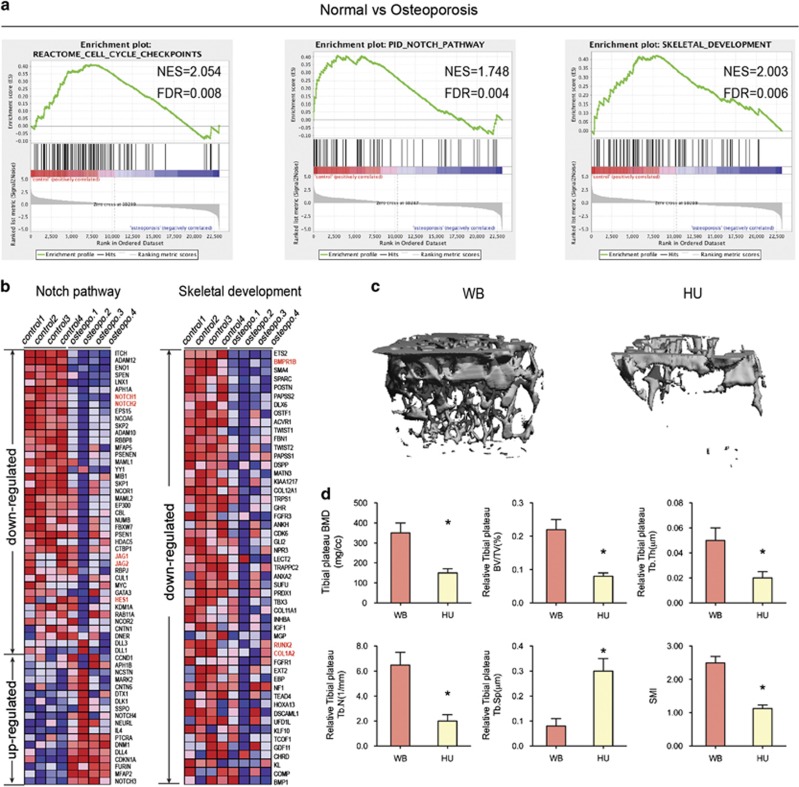
Mechanical loading positively regulates bone formation *in vivo*. (**a**) GSEA of expression profiles of BMSCs from normal (control) and osteoporosis patients. Enrichment curves computed by GSEA are shown in green (FDR-corrected *P*<0.05). GSEA for cell cycle checkpoints, Notch signaling pathway, and skeletal development gene sets demonstrated significant enrichment in control human BMSCs as compared with BMSCs from osteoporosis patients. (**b**) The heat map is ordered by degree of differential expression of Notch signaling pathway and skeletal development genes between BMSCs from normal (control) and osteoporosis patients. (**c**) Representative microCT reconstructive images of tibial plateau of WB and HU mice. *n*=6. (**d**) Three-dimensional microstructural parameters of tibial plateau of WB and HU mice. Data were mean±S.D., *P<0.01. All *P*-values are based on Student's *t*-test

In this study, we compared the transcriptomes of BMSCs from four patients (aged 79–94 years) suffering from primary osteoporosis with the transcriptomes of BMSCs from an age-matched control group (BMSCs-old; donor age, 79–89 years). Genome-wide gene expression patterns were examined using microarray hybridizations (The microarrays were downloaded from the GEO database, GEO accession number GSE35958). Using the GSEA method, we found that the cell cycle checkpoint, Notch signaling pathway, and skeletal development gene sets were significantly enriched in the BMSCs from the control group (the NES scores and FDR values for the gene sets were 2.054, 0.008; 1.748, 0.004; and 2.003, 0.06, respectively) compared with the results from the primary osteoporosis group (Figure 1a). By generating a heat map for gene products that were differentially expressed by at least twofold in the BMSCs from the osteoporosis group relative to their expression in the BMSCs from the age-matched control group, we were able to highlight the differences between BMSCs from the osteoporosis and control groups (Figure 1b). Osteoporotic cells exhibited a distinct gene expression profile independent of cellular aging, in which the components from the NOTCH signaling pathway, JAG1, JAG2, NOTCH1, and NOTCH2, were downregulated, and the expression of their target gene, HES1, was also decreased. As skeletal development or bone formation activity, the markers of osteogenic differentiation of BMSCs, such as RUNX2, COL1a2, and BMPR1B, were decreased in the osteoporosis group. The expression of genes coding for enhancers of osteoblast differentiation and matrix mineralization (SPP1, ALPL, EFNB2, COL1A1, and ANKH) was also reduced (data shown by Benisch *et al.*^24^). These results show that osteogenic differentiation and the related NOTCH signaling were impaired in the BMSCs of patients with osteoporosis.

To investigate the effects of mechanical loading that primarily regulate bone development *in vivo*, we adopted the HU mouse model, which has been widely used to simulate weightlessness and to study various aspects of musculoskeletal loading. For comparison with the effects of WB in the control group mice (WB, 6-month-old age-matched adult mice), HU mice were elevated by their tail for 28 days. Microcomputed tomography (microCT) showed that the bone volume of the tibial plateau in HU mice was significantly lower than that in WB mice (Figures 1c and d).

### Mechanical stimulation orchestrates NOTCH signaling and HDACs in the osteogenic differentiation of BMSCs

**Figure 2:**
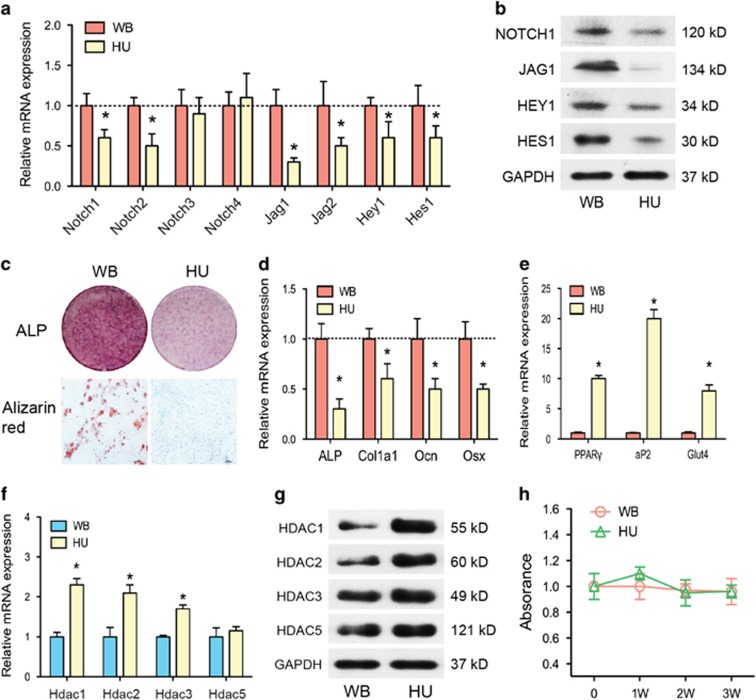
Notch signaling pathways and osteogenic differentiation were impaired in BMSCs of HU mice. (**a**) qRT-PCR analysis of Notch1–4, Jag1, Jag2, Hes1, and Hey1 mRNA levels in BMSCs of WB and HU mice. (**b**) Western blotting analysis of NOTCH1, JAG1, HES1, and HEY1 protein levels in BMSCs of WB and HU mice. (**c**) Representative images of ALP staining and Alizarin red staining of BMSCs from WB and HU mice. Scale bar, 100 *μ*m. (**d**) qRT-PCR analysis of osteogenic differentiation markers Alp, Col1a1, Ocn, and Osx in BMSCs of WB and HU mice. (**e**) qRT-PCR analysis of adipogenic differentiation markers PPAR*γ*, aP2, and Glut4 in BMSCs of WB and HU mice. (**f**) qRT-PCR analysis and (**g**) western blotting analysis of HDACs in BMSCs of WB and HU mice. GAPDH was used as an internal control. (**h**) Cell viability was examined by MTT assay between BMSCs of WB and HU mice. All results are representative of at least three independent experiments. All the staining data were confirmed by three repeated tests. Data were mean±S.D., *P<0.01. All *P*-values are based on Student's *t*-test

We further analyzed the expression of Jag1-Notch signaling and Hdac genes in the BMSCs of the HU group. By isolating BMSCs from the WB and HU groups, we found that, without mechanical loading, the mRNA and protein expression of Notch1, Notch2, Jag1, Jag2, and their target genes Hes1 and Hey1 were all downregulated (Figures 2a and b). Corresponding to the reduced Notch signaling, ALP staining and *in vitro* mineralization were also decreased in the HU group (Figure 2c). These results show that osteogenic differentiation was diminished in the absence of mechanical stimulation. Although the markers of osteogenic differentiation (Alp, Ocn, collagen type I, alpha 1 (Col1a1), and Osx) were downregulated, the markers of adipogenic differentiation (PPAR*γ* (peroxisome proliferator-activated receptor *γ*), aP2, Glut4) were significantly upregulated (Figures 2d and e) in the HU group. The mRNA and protein expression of class I Hdacs (Hdac1, Hdac2, Hdac3) was enhanced, but the expression of class II Hdacs (Hdac5) was not (Figures 2f and g). However, no evident differences in proliferation were found between the two groups after mechanical loading/unloading for 3 weeks in our study (Figure 2h).

**Figure 3:**
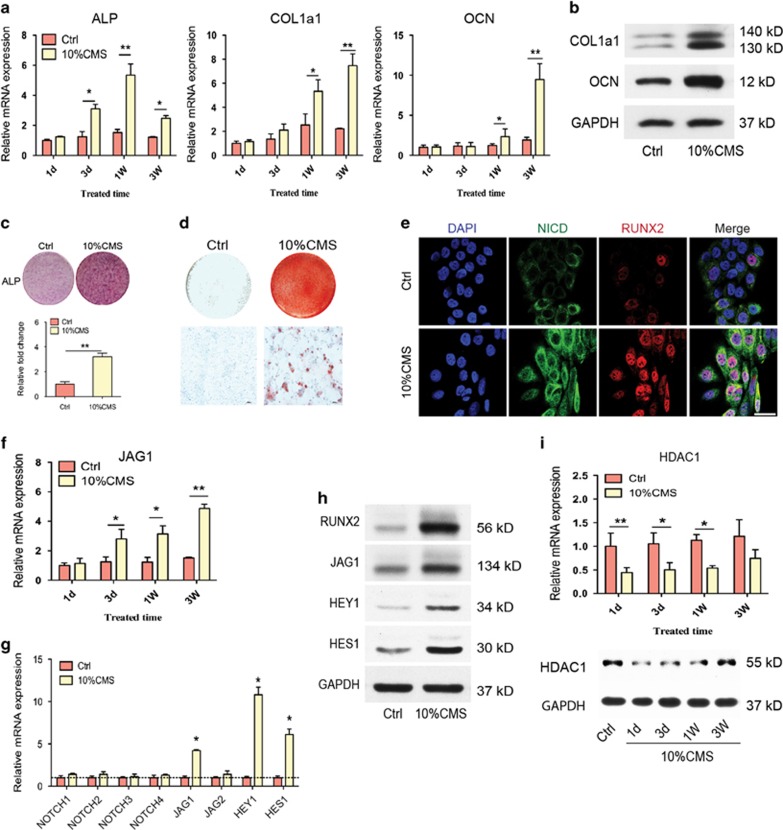
Cyclic mechanical loading could regulate osteogenic differentiation and NOTCH signaling in human BMSCs. (**a**) qRT-PCR analysis and (**b**) western blotting analysis of osteogenic differentiation markers ALP, COL1a1, and OCN in BMSCs after treatment with 10% CMS for 3 weeks compared with static control cells. GAPDH was used as an internal control. (**c**) Representative images of ALP staining (including quantitative analysis) and (**d**) Alizarin red staining of BMSCs after treatment with 10% CMS for 3 weeks compared with static control cells. (**e**) Immunostaining of NICD (green) and RUNX2 (red) location in BMSCs after treatment with 10% CMS for 3 weeks compared with static control cells. Scale bar, 50 *μ*m. (**f**) qRT-PCR analysis of JAG1 expression in BMSCs after treatment with 10% CMS for 3 weeks compared with static control cells. (**g**) qRT-PCR analysis of NOTCH1~4, JAG1, JAG1, HES1, and HEY1 mRNA levels and (**h**) western blot analysis of BMSCs after treatment with 10% CMS for 3 weeks compared with static control cells. (**i**) qRT-PCR analysis and western blotting analysis of HDAC1 in BMSCs after treatment with 10% CMS for 3 weeks compared with static control cells. All results are representative of at least three independent experiments. All the staining data were confirmed by three repeated tests. Data were mean±S.D., *P<0.01, **P<0.001. All *P*-values are based on Student's *t*-test

**Figure 4:**
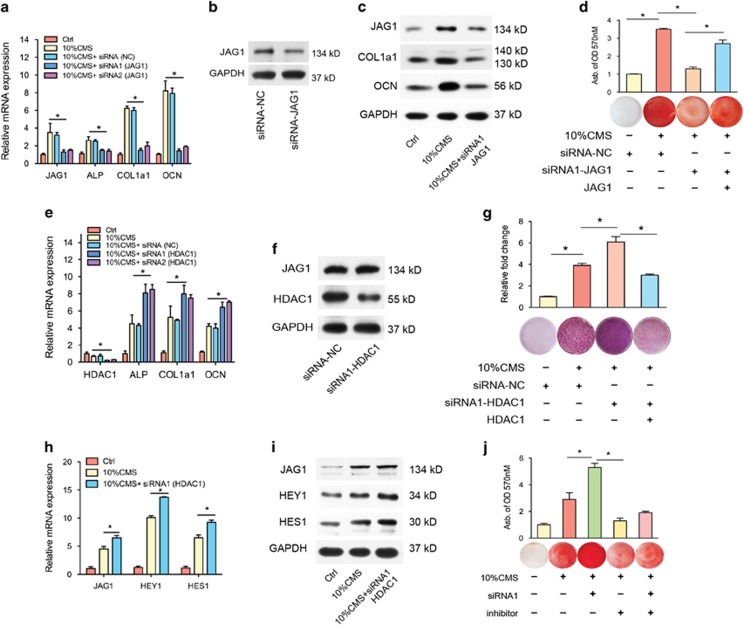
HDAC1 inhibition promoted CMS-induced osteogenic differentiation in human BMSCs. (**a**) qRT-PCR analysis and (**b**, **c**) western blotting analysis of JAG1, ALP, COL1a1, and OCN in BMSCs after transfected with specific siRNA-JAG1 or its corresponding scrambled control (siRNA-NC) under 10% CMS for 3 weeks. Two specific siRNAs for JAG1 were used, and then after the confirmation of knockdown efficiency, siRNA1 was used for the subsequent experiments. (**d**) Alizarin red staining of BMSCs after transfected with specific siRNA-JAG1 or its corresponding scrambled control or JAG1-overexpressing vector under 10% CMS for 3 weeks. (**e**) qRT-PCR analysis of HDAC1, ALP, COL1a1, and OCN in BMSCs after transfected with specific siRNA-HDAC1 or its corresponding scrambled control (siRNA-NC) under 10% CMS for 3 weeks. Two specific siRNAs for HDAC1 were used, and then after the confirmation of knockdown efficiency, siRNA1 was used for the subsequent experiments. (**f**) The knockdown efficiency of HDAC1-specific siRNA (siRNA-HDAC1) was confirmed by comparison to a scrambled control siRNA (siRNA-NC). (**g**) Representative images of ALP staining (including quantitative analysis) of BMSCs after transfected with specific siRNA-HDAC1 or its corresponding scrambled control or HDAC1-overexpressing vector under 10% CMS for 3 weeks. (**h**) qRT-PCR analysis of JAG1, HES1, and HEY1 mRNA levels and (**i**) western blotting analysis of BMSCs after transfected with specific siRNA1-HDAC1 under 10% CMS for 3 weeks. (**j**) Alizarin red staining of BMSCs after co-transfected with specific siRNA-HDAC1 with inhibitor of Notch signaling transduction (10 nM RO4929097) under 10% CMS for 3 weeks. GAPDH was used as an internal control. All results are representative of at least three independent experiments. All the staining data were confirmed by three repeated tests. Data were mean±S.D., *P<0.01. All *P*-values are based on Student's *t*-test

To investigate how mechanical loading regulates osteogenic differentiation *in vitro*, we developed the CMS-induced osteogenic differentiation model of human BMSCs. After loading for 3 weeks, qRT-PCR analysis showed that the expression of the osteogenic marker genes ALP, OCN, and COL1a1 was increased in the CMS group compared with their expression in the non-loading control cells (Ctrl group) (Figure 3a), which was confirmed by the results of the western blotting analysis (Figure 3b). Consistent with the above changes, 10% CMS treatment also enhanced ALP staining (Figure 3c) and *in vitro* mineralization, as assessed by Alizarin red staining of mineralized deposits in the extracellular matrix (Figure 3d). These results show that CMS treatment enhances the osteogenic differentiation of BMSCs. Using immunofluorescence, we found an increased expression of Notch intracellular domain (NICD) and nucleus-accumulated RUNX2 after CMS for 3 days, which indicates the activation of NOTCH signaling and osteogenic differentiation (Figure 3e). Moreover, CMS also promoted the mRNA and protein expression of the ligand for the NOTCH receptor JAG1 and the downstream genes of the NOTCH signaling pathway HES1 and HEY1 (Figures 3f–h). The protein and mRNA levels of HDAC1 were markedly decreased after CMS for 2 weeks, but no significant difference was observed after CMS for 3 weeks (Figure 3i). To confirm the role of JAG1 in osteogenesis, we inhibited JAG1 using a specific siRNA in the CMS-induced osteogenic differentiation model in human BMSCs, and we found that the CMS-induced mRNA and protein expression of JAG1 and the osteogenic markers COL1a1 and OCN were blocked by JAG1 siRNA (Figures 4a–c). The CMS-promoted osteogenic differentiation of BMSCs was also reduced by JAG1 inhibition, as shown by the *in vitro* mineralization results (Figure 4d). All of our results demonstrate that mechanical stimulation promotes the osteogenic differentiation of BMSCs by activating JAG1-mediated pro-osteogenic Notch signaling and reducing the expression of HDAC1.

### HDAC1 modulates JAG1-Notch signaling during CMS-induced osteogenic differentiation

**Figure 5:**
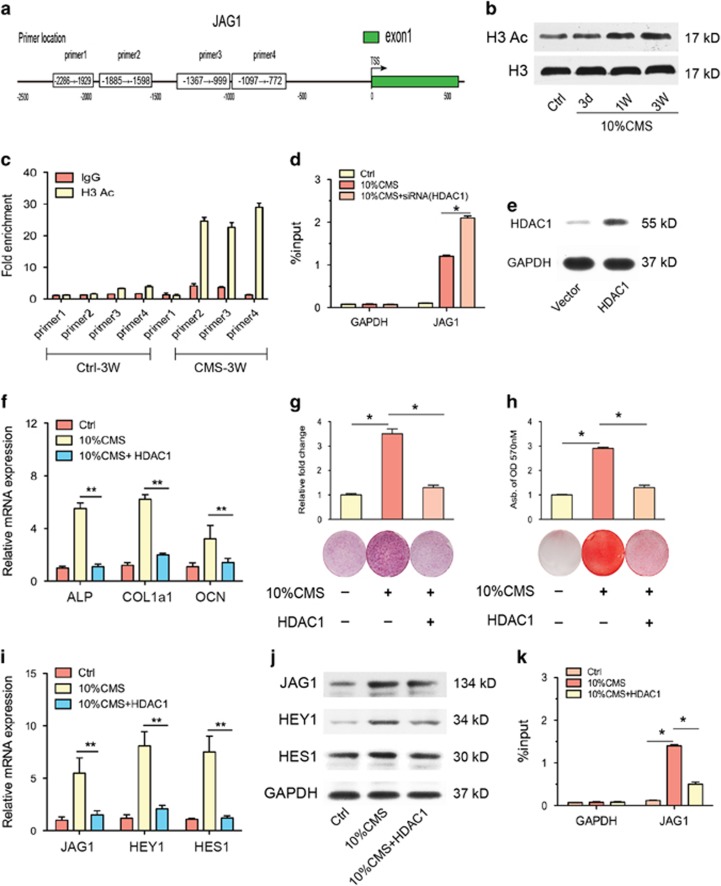
HDAC1 overexpression blocked CMS-induced osteogenic differentiation in human BMSCs. (**a**) Scheme of primers' location in the 5′-flank promoter region of JAG1 gene. The transcriptional start site (TSS) is indicated as +1. (**b**) Western blotting analysis revealed a increase in acetylation of histone H3 (H3) in BMSCs after treatment with 10% CMS for 3 weeks compared with static control cells. (**c**) ChIP-qPCR analysis of H3 acetylation modification in different JAG1 promoter regions in BMSCs after treatment with 10% CMS for 3 weeks compared with static control cells. (**d**) ChIP-qPCR assay on GAPDH and JAG1 promoters. ChIP analysis revealed that there was a significant increase in histone H3 acetylation at JAG1 promoters after siRNA-HDAC1 treatment in BMSCs. (**e**) The efficiency of HDAC1 overexpression was confirmed by comparison to a empty vector. (**f**) qRT-PCR analysis of osteogenic differentiation markers ALP, COL1a1, and OCN. (**g**) ALP staining (including quantitative analysis) and (**h**) Alizarin red staining (including quantitative analysis) in BMSCs after transfected with HDAC1 overexpression or its corresponding negative control under 10% CMS for 3 weeks. (**i**) qRT-PCR analysis of JAG1, HES1, and HEY1 mRNA levels and (**j**) western blotting analysis of BMSCs after transfected with HDAC1 overexpression under 10% CMS for 3 weeks. (**k**) ChIP-qPCR analysis of H3 acetylation modification in different JAG1 promoter regions in BMSCs after transfected with HDAC1 overexpression under 10% CMS for 3 weeks. GAPDH was used as an internal control. All results are representative of at least three independent experiments. All the staining data were confirmed by three repeated tests. Data were mean±S.D., *P<0.01. All *P*-values are based on Student's *t*-test

Next we examined whether there was a connection between the increased JAG1 expression and the decreased HDAC1 expression during CMS-induced osteogenesis, as acetylation of histones has been implicated in the activation of transcription. Previous studies have shown that suppression of HDAC activity using HDAC inhibitors accelerates osteogenesis.^20^ To clarify the role of HDAC1 in regulating CMS-induced osteogenesis, we treated human BMSCs with an HDAC1 inhibitor or caused them to overexpress HDAC1. First, we inhibited HDAC1 using a specific siRNA in the CMS-induced osteogenic differentiation model. Without effecting JAG1 expression (Figures 4e and f), we found that the CMS-induced expression of the osteogenic markers ALP, COL1a1, and OCN were enhanced by HDAC1 siRNA treatment, which was further supported by the ALP staining results (Figure 4g). Furthermore, we investigated the influence of HDAC1 on the expression of the JAG1-NOTCH signaling pathway and, therefore, on osteogenesis. We found that the CMS-activated mRNA and protein expression of JAG1, HES1, and HEY1 were facilitated by HDAC1 inhibition (Figures 4h and i). Interestingly, as shown by the *in vitro* mineralization assay, CMS-activated osteogenesis was enhanced by HDAC1 inhibition. However, this enhancement by HDAC1 inhibition was significantly blocked by an inhibitor of NOTCH signaling transduction (10 nM RO4929097, an inhibitor of *γ* secretase) (Figure 4j). Moreover, we then investigated whether the observed pro-osteogenic effects of HDAC1 inhibition were related to changes in histone acetylation in the promoter regions in human BMSCs. A ChIP analysis was performed using antibodies to pan-acetylated histone H3 and four designed primers for JAG1 promoters (Figure 5a). We identified a significant increase in the histone H3 acetylation level at the JAG1 promoter after CMS treatment for 3 weeks (Figures 5b and c). HDAC1 inhibition promoted the elevated H3 acetylation level at the JAG1 promoter (Figure 5d).

Second, we overexpressed HDAC1 in human BMSCs (Figure 5e) and found that this significantly reduced ALP and COL1a1 transcription and the level of the late osteoblast marker OCN under mechanical stimulation conditions (Figure 5f). ALP assays and Alizarin red staining for mineralized deposits showed that HDAC1 overexpression significantly abrogated the CMS-induced increase in ALP activity and mineralized deposits (Figures 5g and h). Furthermore, the CMS-induced expression of JAG1, HES1, and HEY1 were also blocked by HDAC1 overexpression (Figures 5i and j). Consistent with the above results, the CMS-enhanced histone H3 acetylation level at the JAG1 promoter was apparently decreased by the induction of HDAC1 overexpression (Figure 5k). Overall, these results confirm that CMS can induce osteogenic differentiation of BMSCs by activating pro-osteogenic JAG1-Notch signaling, which is facilitated by increased histone H3 acetylation levels.

### Inhibition of HDAC1 rescued the decrease of bone formation in the HU mouse model

**Figure 6:**
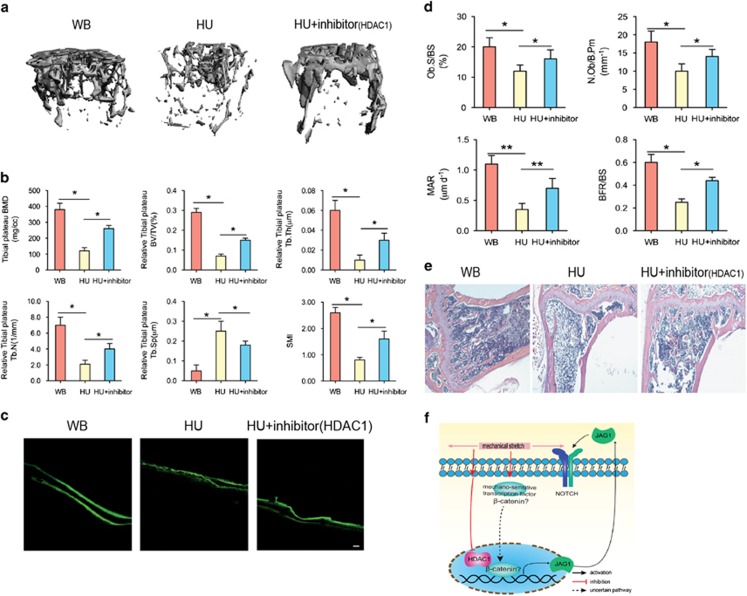
Inhibition of HDAC1 rescued the decrease of bone formation *in vivo*. (**a**) Representative microCT reconstructive images of tibial plateau in WB, HU, and HU+HDAC1 inhibitor mice. *n*=6. (**b**) Three-dimensional microstructural parameters of tibial plateau in WB, HU, and HU+HDAC1 inhibitor mice. (**c**) Representative images showing new bone formation assessed by double calcein labeling in each group. *n*=4. Scale bars, 50 *μ*m. (**d**) Histomorphometric analysis of bone formation–related parameters (Ob.S/BS, MAR, BFR, and N.Ob/B.Pm) in WB, HU, and HU+HDAC1 inhibitor mice. (**e**) Representative hematoxylin–eosin staining images of tibial plateau showing bone volume in each group. *n*=6. (**f**) Schematic diagram of the role of HDAC1 in regulating MSC differentiation and bone formation under mechanical stimulation. Data were mean±S.D., *P<0.01. All *P*-values are based on Student's *t-*test

To investigate the function of HDAC1 *in vivo*, we used a HU mouse model. MicroCT showed that mechanical unloading-induced bone loss was partly rescued by HDAC1 inhibition (valproic acid) (Figure 6a), and bone volume analysis revealed that the bone volume-related parameters were partly increased by HDAC1 inhibition (Figure 6b). Similarly, the assessment of bone formation indicated by green fluorescent calcein showed that the mechanical unloading-induced decrease in bone formation was rescued by HDAC1 inhibition (Figures 6c and d). Furthermore, a histological assessment after 28 days of HU demonstrated that, compared with staining in the WB group, the bone staining of the HU group was significantly decreased, with intensified adipose tissue staining. The overall area of stained bone was increased in the HU group treated with an HDAC1 inhibitor, which indicates that the impaired development of new bone was rescued by HDAC1 inhibition (Figure 6e). Taken together, these results indicate that osteogenic differentiation and Notch signaling are impaired in the BMSCs of patients with osteoporosis and in HU mice. In addition, mechanical stimulation promotes the osteogenic differentiation of BMSCs by, at least in part, activating JAG1-mediated pro-osteogenic Notch signaling and reducing the expression of HDAC1. Finally, the therapeutic inhibition of HDAC1 was able to partly counteract the bone loss observed in HU mice (Figure 6f).

## Discussion

Understanding how MSCs sense and respond to applied forces is an area of intense research. Numerous cell membrane proteins have been identified in mechano-sensing mechanisms.^25, 26, 27^ Cadherins, which bind cells to adjacent cells, have also been found to anchor themselves by forming complexes with catenins, which bind directly to the cytoskeleton. Mechanical loading inhibits the binding of *β*-catenin to E-cadherin and increases the cytoplasmic pool of *β*-catenin.^28^ The decrease of *β*-catenin binding is coupled with Akt and GSK3*β* activation. Therefore, cadherins may serve as mechano-sensors and may be a promising target for future mechanical loading studies. The expression of Notch, a cell-surface receptor that transduces short-range signals by interacting with transmembrane ligands such as Delta (termed Delta-like in humans) and Serrate (termed Jagged in humans) on neighboring cells, had not yet been studied under mechanical stimulation conditions. Notch signaling is a key mechanism in the control of stem cell differentiation and embryogenesis.^29^ Notch signaling components, especially JAG1 and NOTCH-2, are upregulated during both endochondral and intramembranous bone regeneration.^30^ JAG1 is variably mutated in Alagille syndrome patients with skeletal defects and poor bone healing.^31^ In addition, JAG1 has been identified as a gene associated with osteoporosis in a GWAS analysis, and a recently identified Jag1-null mutation is responsible for the development of osteogenesis imperfecta.^23^ Studies have also found that JAG1-activated Notch signaling is sufficient to induce human MSC osteogenesis, increasing osteogenic marker genes, such as ALP and BSP, and enhancing ALP activity and tissue mineralization.^21^

Despite the clinical and genetic evidence suggesting that JAG1 may positively regulate bone mass in humans, there is a paucity of data from studies exploring the role of JAG1 in human osteogenesis under mechanical loading. For the first time, our results show that Jag1 and Notch signaling were decreased in the BMSCs of hindlimb-unloaded mice and that JAG1-activated NOTCH signaling in human BMSCs was upregulated by CMS *in vitro*. Furthermore, JAG1 and Notch signaling had an important role in mediating the mechanical-triggered osteogenic differentiation of BMSCs, which was blocked by inhibitors of the NOTCH signaling pathways. However, we also observed that CMS modulated JAG1 expression by modifying the histone acetylation status of JAG1, as the inhibition of HDAC1 only partially rescued the mechanical unloading-induced osteoporosis in mice. This suggests that CMS may also directly induce JAG1 expression by activating mechano-sensitive transcription factors, such as *β*-catenin, and change the epigenetic modifications of the JAG1 promoter region to promote its transcription. NOTCH signaling and JAG1 expression were both impaired because of the loss of mechanical loading, but they may also be affected by the loss of miscellaneous biochemical or spatial structural signals that occurs in osteoporosis *in vivo*. This topic deserves in-depth studies to determine how BMSCs orchestrate the biochemical and mechanical signals involved in this process and which signal is the main factor in controlling osteogenic differentiation *in vivo*.

Stem cell differentiation is extremely sensitive to epigenetic changes. The application of epigenetic regulators, such as inhibitors of histone-modification enzymes, may be valuable for stem cell-based interventions.^32^ HDAC inhibitors have been demonstrated to enhance osteogenic differentiation *in vitro* and new bone formation *in vivo*.^19^ Previous studies have shown that the suppression of HDAC activity with HDAC inhibitors accelerates osteogenesis by inducing osteoblast marker genes, including osteopontin and ALP. Moreover, the osteogenesis-promoting effects of VPA on the expression of bone matrix markers are related to changes in histone acetylation in the promoter regions.^33^ It has also been shown that mechanical cues could directly induce changes in epigenetic modifications.^34^ In vascular endothelial cells (ECs), hemodynamic force-induced histone modifications have been extensively studied in recent years. Shear stress can modulate chromatin remodeling on histone H3 and H4, resulting in eNOS being regulated by chromatin-based epigenetic mechanisms at the transcriptional level.^35^ Zeng *et al.*^36^ demonstrated that laminar flow increased the activity of HDACs and the association of p53 with HDAC1, leading to the deacetylation of p53 in ECs. Lee *et al.*^37^ utilized HDAC-specific siRNAs and found that class I HDAC1/2/3, but not class II HDAC4/7, modulated oscillatory flow-induced cell proliferation. However, the potential of the mechanical environment to regulate DNA methylation or histone modifications has seldom been examined in BMSCs, which is fundamental to understanding stem cell mechanobiology. Arnsdorf *et al.*^38^ demonstrated that mechanical stimulation altered the epigenetic state of osteogenic genes (Ocn, Opn, and Col1) by reducing DNA methylation and showed that this was associated with an increase in expression. Zuo *et al.*^13^ found that miR-103a is a mechano-sensitive miRNA that regulates osteoblast differentiation via directly targeting Runx2. For the first time, we found that mechanical loading downregulated HDAC1 and, therefore, facilitated JAG1 expression and the osteogenesis of BMSCs. Furthermore, we also found increased Hdac1 expression and downregulated Jag1-Notch signaling in a mechanical unloading-induced osteoporotic mouse model. The inhibition of Hdac1 by a specific siRNA or small-molecule inhibitor promoted osteogenesis and rescued bone loss in mechanically unloaded mice. Consistent with the above-mentioned reported results, our results confirmed that mechanical cues can directly modulate the epigenetic modification status of osteogenic genes and, therefore, promote the osteogenic differentiation of BMSCs.

Several studies have indicated that mechanical force has an important role in regulating cell growth and proliferation, and an appropriate mechanical stretch treatment could promote the proliferative capacity of BMSCs. Song *et al.*^39^ found that the proliferation of rat bone marrow mesenchymal stem cells was significantly elevated after exposure to a 1-Hz stretch stimulation within 15–60 min at an 8% strain. However, Zuo *et al.*^13^ found that 8% CMS had no significant influence on cell viability during 24–72 h. The effect of long-term (>3 weeks) continuous mechanical stretching on the proliferation of MSCs has not been previously studied. Luu *et al.*^40^ found that 6 weeks of low-magnitude mechanical signals (0.2 g, 90-Hz signal applied for 15 min/day, 5 day/week) increased the overall marrow-based stem cell population by 37% and the number of MSCs by 46%. Concomitant with the increase in stem cell number, the differentiation potential of MSCs in the bone marrow was biased toward osteogenic and against adipogenic differentiation. In our study, we found that without mechanical loading, the proliferation of BMSCs was unchanged after 3 weeks. There are many studies that have shown mechanical stimulation to have no effect on cell proliferation^8, 41^ or to reduce MSC proliferation.^2, 42, 43^ These mixed findings can most likely be explained by the diversity of conditions in the experiments, including the specific mechanical stimulation, MSC species, and culture media used, as well as the wide range of loading parameters used.

## Conclusion

In summary, our study provides new findings that mechanical stimulation orchestrates gene expression for the osteogenic differentiation of BMSCs via directly regulating HDAC1. HDAC1 functions through inhibiting its direct target, JAG1, which is the master regulator of osteogenesis, at the transcription level. Understanding the molecular mechanisms of epigenetic modifiers such as HDACs in regulating MSC lineage determination under mechanical stimulation is pivotal for understanding bone cell differentiation and diseases. These findings not only provide new insights into mechano-response signaling pathways but also raise intriguing possibilities for the use of HDAC modulators to regulate bone formation in regenerative medicine. We anticipate that our study will provide a foundation for future investigations on the development of gene therapies for treating human bone remodeling disorders related to mechanical loading, such as osteoporosis.

## Materials and Methods

### Cell culture

Bone marrow cells from the tibias and femurs of mice and the posterior iliac crests of healthy adult human donors (17–35 years of age), collected with informed consent, were flushed out with *α*-Minimum Essential Medium (*α*-MEM, Hyclone, Logan, UT, USA) and cultured in growth medium (*α*-MEM with 10% fetal bovine serum (Gibco by Invitrogen, Carlsbad, CA, USA) with 1% penicillin and streptomycin (Hyclone)) at 37° in the presence of 5% CO_2_ following the lysis of red blood cells. Non-adherent cells were removed by replacing the medium after 3 days. The attached BMSCs were used for experiments at passages 3–5.

For the transfection of siRNA oligos, the cells in the culture medium were transfected using Lipofectamine 2000 transfection reagent (Invitrogen, Carlsbad, CA, USA), which was used according to the manufacturer's instructions. The siRNA was transfected at a concentration of 50 nM. The siRNA sequences used in this study were as follows: for JAG1, siRNA1: 5′-AGGCTGCGCATAATCATAATA-3′, siRNA2: 5′-GGCTGCGCATAATCATAATAA-3′; and for HDAC1, siRNA1: 5′-GGAGGAAAGTCTGTTACTACT-3′, siRNA2: 5′-GAGGAAAGTCTGTTACTACTA-3′.

### Hindlimb unloading (HU) in mice

Six-month-old male C57BL/6J mice were purchased from Shanghai SLAC Laboratory Animal Co. Ltd, Shanghai, China. The animals were suspended from the hindlimbs for a period of 28 days, as previously described.^13^ All of the experimental procedures were approved by the Committees of Animal Ethics and Experimental Safety of Shanghai Ninth People's Hospital.

### Retroviral transduction overexpression studies

Human JAG1 and HDAC1 genes were ligated into pRUF-IRES-GFP using PCR primers to amplify the coding region. The pRUF- IRES-GFP and pRUF-IRES-GFP-JAG1 and HDAC1 constructs were transfected into the HEK 293T viral packaging cell line together with the Pol and GAG protein (PGP) and vesicular stomatitis virus G-protein (VSVG) (viral envelope proteins, SBI System Biosciences, Mountain View, CA, USA). The viral supernatant was used for the infection of MSCs as previously described.^44^ Stable lines were generated by sorting for GFP-positive cells using fluorescence-activated cell sorting.

### Microarray analysis and Gene Set Enrichment Analysis (GSEA)

The microarrays for human BMSCs of elderly individuals or patients with osteoporosis (GSE35959) were obtained from the GEO database. To identify genes that are differentially expressed between normal and osteoporosis donors, the spot intensity data of all relevant samples were analyzed using GeneSight-Lite 4.1.6 (BioDiscovery, EI Segundo, CA, USA). Normalization of the expression profiles was performed by dividing values by the mean signal of each array representing a single sample. The resulting data were visualized using the Multiple Experiment Viewer application (Boston, MA, USA). Gene sets from the complete C2 curated gene sets and C5 GO gene sets, downloaded from Molecular Signatures Database (MSigDB), were tested for enrichment against the human osteoporosis phenotype based on the GSEA method.^45^ The complete series of human BMSCs in samples from four elderly donors (GSM878100, GSM878101, GSM878102, and GSM878103) and four osteoporosis donors (GSM878104, GSM878105, GSM878106, and GSM878107) were used for this analysis. Genes were sorted according to the value of the *t*-statistic computed against the human BMSC ‘Normal versus Osteoporosis' phenotype, with genes upregulated in the ‘Normal' class at the left-end of the list and genes upregulated in the ‘Osteoporosis' class at the right-end of the list; the cell cycle checkpoint, Notch pathway and skeletal development genes were located within the sorted list, and their position was determined to be significantly skewed toward the ‘Normal' end of the sorted list based on a weighted Kolmogorov–Smirnoff test. The resulting heat map and the intensity data were partly inspected for genes differentially expressed between the BMSCs of normal donors and those with osteoporosis.

### Cyclic mechanical stretch application

BMSCs were plated at a density of 5 × 10^5^ cells/cm^2^ (if not mentioned) in 1 ml of medium on six-well flexible silicone rubber BioFlex plates coated with collagen type I (Flexcell International Corporation, Hillsborough, NC, USA). Cells were cultured for 24 h to reach 50–60% confluency before mechanical tension was applied, which guaranteed sufficient space for cell proliferation and an adequate number of cells for the following experiments. Cyclic mechanical stretch (CMS) with a 0.5-Hz sinusoidal curve at 10% elongation was applied using an FX-5000 T Flexercell Tension Plus unit (Flexcell International Corporation). The cultures were incubated in a humidified atmosphere at 37 °C and 5% CO_2_ during the stretching. Cells were harvested immediately after the application of CMS stimulation was completed. Control cells were cultured on the same plates in the same incubator but were not subjected to stretching.

### Proliferation assays

For the proliferation assays, BMSCs were seeded at a density of 4000 cells/well in 96-well plates, and cell proliferation was monitored after the indicated time points using an MTT (3-(4,5-dimethylthiazol-2-yl)-2,5-diphenyltetrazolium bromide) assay.

### ALP staining

ALP staining was performed on cultured cells. The cell layer was rinsed with phosphate-buffered saline (PBS) three times, followed by fixation in 4% paraformaldehyde for 10 min at room temperature. The cells were then incubated with buffer containing 0.1% naphthol AS-TR phosphate and 2% fast violet B (Sigma-Aldrich, St Louis, MO, USA). After incubation for 1 h at 37 °C, the cell layer was washed with deionized water.

### Alizarin red staining

Cells were fixed in 70% ice-cold ethanol for 1 h and rinsed with double-distilled H_2_O (ddH_2_O). Cells were then stained with 40 mM Alizarin red S (Sigma, St. Louis, MO, USA), pH 4.0, for 15 min with gentle agitation. After staining, cells were rinsed five times with ddH_2_O. For the quantitative assessment of the degree of mineralization, the red stain was eluted by 10% (w/v) cetylpyridinium chloride (Sigma-Aldrich) for 1 h and quantified via spectrophotometric absorbance measurements of optical density at 570 nm.

### RNA purification and quantitative real-time PCR (qRT-PCR)

The total RNA of cells was isolated using TRIzol reagent (Invitrogen) according to the manufacturer's instructions. After the reverse transcription reaction, RT-PCR was performed with an ABI 7900HT system using SYBR Premix (Takara, Dalian, China) according to the manufacturer's instructions. The conditions of the RT-PCR were as follows: denaturation at 95 °C for 10 s, 40 cycles at 95 °C for 10 s, and 60 °C for 30 s. A dissociation stage was added at the end of the amplification procedure. No nonspecific amplification was observed, as determined using the dissociation curve. Glyceraldehyde 3-phosphate dehydrogenase (GAPDH) was used as an internal control. The data were analyzed using the comparison Ct (2^−ΔΔCt^) method and expressed as the fold change relative to the respective control. Each sample was analyzed in triplicate. The primer sequences used in this study were as follows: GAPDH: forward, 5′-CCTCTGACTTCAACAGCGAC-3′ reverse, 5′-TCCTCTTGTGCTCTTGCTGG-3′ ALP: forward, 5′-GAGTCGGACGTGTACCGGA-3′ reverse, 5′-TGCCACTCCCACATTTGTCAC-3′ runt-related transcription factor 2 (RUNX2): forward, 5′-GCCTTCAAGGTGGTAGCCC-3′ reverse, 5′-CGTTACCCGCCATGACAGTA-3′ COL1a1: forward, 5′-CAGCCGCTTCACCTACAGC-3′ reverse, 5′-TTTTGTATTCAATCACTGTCTTGCC-3′ OCN: forward, 5′-GAAGCCCAGCGGTGCA-3′ reverse, 5′-CACTACCTCGCTGCCCTCC-3′ OSX: forward, 5′-CCCTTCTCAAGCACCAATGG-3′ reverse, 5′-AAGGGTGGGTAGTCATTTGCATA-3′ fatty acid-binding protein-4 (aP2): forward, 5′-AAATCACCGCAGACGACA-3′ reverse, 5′-CACATTCCACCACCAGCT-3′ glucose transporter type 4 (Glut4): forward, 5′-CTTGGCTCCCTTCAGTTTG-3′ reverse, 5′-TGCCTTGTGGGATGGAAT-3′ hes family bHLH transcription factor 1 (HES1): forward, 5′-TCAACACGACACCGGATAAAC-3′ r1verse, 5′-GCCGCGAGCTATCTTTCTTCA-3′ hes-related family bHLH transcription factor with YRPW motif 1 (HEY1): forward, 5′-GTTCGGCTCTAGGTTCCATGT-3′ reverse, 5′-CGTCGGCGCTTCTCAATTATTC-3′ for human: JAG1: forward, 5′-GTCCATGCAGAACGTGAACG-3′ reverse, 5′-GCGGGACTGATACTCCTTGA-3′ JAG2: forward, 5′-TGGGCGGCAACTCCTTCTA-3′ reverse, 5′-GCCTCCACGATGAGGGTAAA-3′ NOTCH1: forward, 5′-GAGGCGTGGCAGACTATGC-3′ reverse, 5′-CTTGTACTCCGTCAGCGTGA-3′ NOTCH2: forward, 5′-CCTTCCACTGTGAGTGTCTGA-3′ reverse, 5′-AGGTAGCATCATTCTGGCAGG-3′ NOTCH3: forward, 5′-CGTGGCTTCTTTCTACTGTGC-3′ reverse, 5′-CGTTCACCGGATTTGTGTCAC-3′ NOTCH4: forward, 5′-TGTGAACGTGATGTCAACGAG-3′ reverse, 5′-ACAGTCTGGGCCTATGAAACC-3′ HDAC1: forward, 5′-CTACTACGACGGGGATGTTGG-3′ reverse, 5′-GAGTCATGCGGATTCGGTGAG-3′ for mice: Notch1: forward, 5′-GATGGCCTCAATGGGTACAAG-3′ reverse, 5′-TCGTTGTTGTTGATGTCACAGT-3′ Notch2: forward, 5′-GAGAAAAACCGCTGTCAGAATGG-3′ reverse, 5′-GGTGGAGTATTGGCAGTCCTC-3′ Notch3: forward, 5′-AGTGCCGATCTGGTACAACTT-3′ reverse, 5′-CACTACGGGGTTCTCACACA-3′ Notch4: forward, 5′-CCCCGGAGCATTCTTCTGC-3′ reverse, 5′-AGTCCAGCCCTCATCACACA-3′ Jag1: forward, 5′-ATGCAGAACGTGAATGGAGAG-3′ reverse, 5′-GCGGGACTGATACTCCTTGAG-3′ Jag2: forward, 5′-TTCTGTGACGAGTGTGTCCC-3′ reverse, 5′-GCGCAGAGGTATTGGTCAGG-3′ Hdac1: forward, 5′-TGAAGCCTCACCGAATCCG-3′ reverse, 5′-GGGCGAATAGAACGCAGGA-3′ Hdac2: forward, 5′-GGAGGAGGCTACACAATCCG-3′ reverse, 5′-TCTGGAGTGTTCTGGTTTGTCA-3′ Hdac3: forward, 5′-GCCAAGACCGTGGCGTATT-3′ reverse, 5′-GTCCAGCTCCATAGTGGAAGT-3′ and Hdac5: forward, 5′-AGCACCGAGGTAAAGCTGAG-3′ reverse, 5′-GAACTCTGGTCCAAAGAAGCG-3′.

### Western blotting analysis

For the western blotting analysis, cells were lysed on ice for 30 min in a lysis buffer containing 50 mM Tris–HCl (pH 7.4), 150 mM NaCl, 1% Nonidet P-40, and 0.1% SDS supplemented with protease inhibitors (10 mg/ml leupeptin, 10 mg/ml pepstatin A, and 10 mg/ml aprotinin). Protein fractions were collected by centrifugation at 15 000 *g* at 4 °C for 10 min and then subjected to 10% SDS-PAGE and transferred to polyvinylidene difluoride membranes. The membranes were blocked with 5% BSA and incubated with specific antibodies overnight at 4 °C. A horseradish peroxidase–labeled secondary antibody was added and visualized using an enhanced chemiluminescence detection system (Millipore, Billerica, MA, USA) as recommended by the manufacturer. We used the following primary antibodies to determine the concentrations of proteins in the lysates: anti-human RUNX2 rabbit mAb, anti-JAG1, HEY1 mAb (1 : 1000, Abcam, Cambridge, UK), anti-NOTCH1, HES1 mAb (1 : 1000, Cell Signaling Technology, Inc., Danvers, MA, USA), GAPDH rabbit mAb (1 : 1000, Cell Signaling Technology, Inc.), anti-HDAC1, HDAC2, HDAC3, HDAC5 mAb (1 : 1000, Abcam), anti-COL1a1, OCN (1 : 500, Abcam), and anti-AcH3 antibody (1 : 1000, Millipore, Bedford, MA, USA)).

### Immunofluorescence

BMSCs cultured in six-well plates were fixed with 4% PFA in PBS for 20 min at room temperature. After washing in PBS, samples were permeabilized with 0.5% Triton X-100 for 5 min and blocked with 5% BSA for 60 min. An incubation with primary anti-NICD and anti-RUNX2 antibodies (Abcam) was performed overnight at 4 °C. The primary antibodies were detected using FITC or PE-conjugated anti-mouse IgG secondary antibodies. After the final wash, the nuclei were counterstained by adding a 2-mg/ml solution of 4′,6-diamidino-2-phenylindole (Sigma-Aldrich) in PBS for 10 min before imaging. Cells were visualized using a confocal microscope (Leica, Solms, Germany).

### Bone histomorphometric analyses

We measured the structure of the tibial plateau with a SCANCO Medical *μ*CT 40 scanner. The images were analyzed using the SCANCO evaluation software to perform segmentation, conduct a three-dimensional morphometric analysis, and determine the density and distance parameters (SCANCO Medical AG, Zurich, Switzerland). The three-dimensional structural parameters analyzed included the following: TV (total tissue volume, containing both trabecular and cortical bone), BV/TV (trabecular bone volume per tissue volume), Tb.Th (trabecular thickness), Tb.Sp (trabecular separation), and SMI (structure model index). For the assessment of new bone formation, we injected green fluorescent calcein (Sigma; 5 mg/kg body weight) into the mice on days 7 and 2 before killing. Bone histomorphometric analyses for OB number per bone surface (Ob.S/BS), OB number per bone perimeter (N.Ob/B.Pm), bone formation rate/bone surface (BFR/BS), and mineral apposition rate (MAR) were performed using the professional image analysis software (Image J; NIH, Bethesda, MD, USA) under fluorescence microscopy (Leica, Q500MC). The bone histomorphometric parameters were calculated and expressed according to the standardized nomenclature for bone histomorphometry.

### Chromatin immunoprecipitation (ChIP) assay

ChIP assays were performed using an EZ ChIP Chromatin Immunoprecipitation Kit (Millipore, Upstate, NY, USA) according to the manufacturer's instructions. Briefly, cells cultured under the previously indicated conditions were fixed in 1% formaldehyde/PBS for 10 min at room temperature. After two washes with PBS, cells were resuspended in 0.5 ml of lysis buffer containing a protease inhibitor cocktail before sonication. DNA fragments from the soluble chromatin preparations were 400–800 bp in length. Immunoprecipitation was carried out overnight with purified anti-AcH3 antibody (Millipore, Bedford, MA, USA) or normal mouse IgG as a negative control. Protein A/G agarose was used to pulldown the antigen–antibody compounds and then washed four times with washing buffers. The DNA–protein crosslinks were reversed with 5 M NaCl at 65 °C for 6 h, and DNA from each sample was purified. PCR was performed using 2 *μ*l DNA samples with the following primers: JAG1 primer 1: forward, 5′-TTCTAGGTGAAGCCAGGTGGAG-3′ reverse, 5′-AATACAAAAATTAGCTGGGCGTG-3′ primer 2: forward, 5′-AATCTCTTGACCTCGTGATCCACC-3′ reverse, 5′-AGCGACAACCTGGGTGTTTCAAT-3′ primer 3: forward, 5′-GAATGATGAGATTTGGCACTGAA-3′ reverse, 5′-CTGGTCATAATCAAGGTCGAAGA-3′ and primer 4: forward, 5′-TATAAAGGTCCCCTCAAATGCAAC-3′ reverse, 5′-AGATGCTGGTGGGCTTGGAC-3′.

### Therapeutic inhibition of HDAC1 in HU mice

Six-month-old C57BL/6J mice received tail-vein injections of valproic acid (HU+inhibitor group, valproic acid was purchased from Selleckchem (Shanghai, China), 50 mg/kg body weight, 0.2 ml per injection) twice a week for 2 weeks or no treatment (HU group). The mice were subjected to HU via tail suspension for 27 days and were then killed. Tissues were harvested, and measurements of bone formation in the tissues were performed.

### Statistical analysis

The data are presented as the mean±S.D. (*n* is the number of tissue preparations, cells, or experimental replicates). For comparing groups of data, a two-tailed Student's *t*-test was used. A value of *P*<0.05 was considered to be statistically significant.

## Abbreviations

BMSC: bone marrow stromal cell
CMS: cyclic mechanical stretch
ChIP: chromatin immunoprecipitation
ALP: alkaline phosphatase
qRT-PCR: quantitative real-time PCR
BMP: bone morphogenetic protein
Osx: osterix
PPAR: peroxisome proliferator-activated receptor
Col: collagen
Ocn: osteocalcin
aP2: fatty acid-binding protein-4
Glut4: glucose transporter type 4
PBS: phosphate-buffered saline
GSEA: Gene Set Enrichment Analysis
HU: hindlimb unloading
WB: weight bearing
H3: histone H3
Ac: acetylation
Runx2: runt-related transcription factor 2
JAG1: jagged 1
Hes1: hes family bHLH transcription factor 1
Hey1: hes-related family bHLH transcription factor with YRPW motif 1
HDAC: histone deacetylase
